# Impact of steatotic liver diseases on diabetes mellitus risk in patients with atrial fibrillation: a nationwide population study

**DOI:** 10.1186/s12933-025-02795-5

**Published:** 2025-06-07

**Authors:** Hyunho Ryu, So-Ryoung Lee, Eue-Keun Choi, Young-Hae Go, Kyung-Yeon Lee, JungMin Choi, Seokmoon Han, Jae-Hyun Kim, Hyo-Jeong Ahn, Soonil Kwon, Bong-Seoung Kim, Kyung-Do Han, Seil Oh, Gregory Y. H. Lip

**Affiliations:** 1 Department of Internal Medicine, Seoul National University Hospital, Seoul, Republic of Korea; 2 Department of Internal Medicine, Seoul National University College of Medicine, 101 Daehak-ro, Jongno-gu, Seoul, 03080 Republic of Korea; 3 Department of Internal Medicine, Seoul Metropolitan Government-Seoul National University Boramae Medical Center, Seoul, Republic of Korea; 4 Department of Statistics and Actuarial Science, Soongsil University, 369 Sangdo-ro, Dongjak-gu, Seoul, 06978 Republic of Korea; 5 Liverpool Centre for Cardiovascular Science at University of Liverpool, Liverpool John Moores University and Liverpool Chest & Heart Hospital, Liverpool, UK; 6 Department of Clinical Medicine, Aalborg University, Aalborg, Denmark

**Keywords:** Atrial fibrillation, Diabetes mellitus, Steatotic liver disease

## Abstract

**Background:**

Atrial fibrillation (AF) frequently coexists with diabetes mellitus (DM), leading to a worse prognosis if both are present. Steatotic liver disease (SLD) may also predispose to DM, but its impact among AF patients is unclear. We aimed to determine whether metabolic dysfunction-associated SLD (MASLD), MASLD with increased alcohol intake (MetALD), or alcohol-related liver disease (ALD) elevates DM risk in AF.

**Methods:**

Non-diabetic individuals who developed AF between 2010 and 2018 from the Korean National Health Insurance Service database were included. Patients with a fatty liver index (FLI) < 30 were classified as non-SLD, whereas those with FLI ≥ 30 and at least one cardiometabolic risk factors were categorized as MASLD, MetALD, or ALD based on daily alcohol intake. Incident DM hazard ratios (HRs) were estimated with Cox regression models.

**Results:**

Among 195,195 patients (mean age 64.4 ± 13.0 years, 57.5% male); 108,918 (55.8%) in non-SLD, 71,795 (36.8%) in MASLD, 7644 (3.9%) in MetALD, and 6838 (3.5%) in ALD, respectively. Over a mean follow-up of 6.0 ± 2.9 years, 25,632 (13.0%) developed DM. Compared with non-SLD, the adjusted HRs with 95% confidence intervals (CIs) for incident DM were 1.930 (1.879–1.983), 1.789 (1.682–1.904), and 1.932 (1.817–2.054) for MASLD, MetALD, and ALD, respectively. In the age 20–39 years group, adjusted HRs with 95% CIs were 5.844 (4.501–7.587), 5.354 (3.681–7.787), and 7.033 (4.660–10.615), respectively.

**Conclusion:**

SLD confers an increased risk of new-onset DM in AF patients, especially in younger adults. Implementing management strategies to prevent DM in AF patients with SLD might mitigate the risk of DM and its potential impact on AF-related outcomes.

**Graphical abstract:**

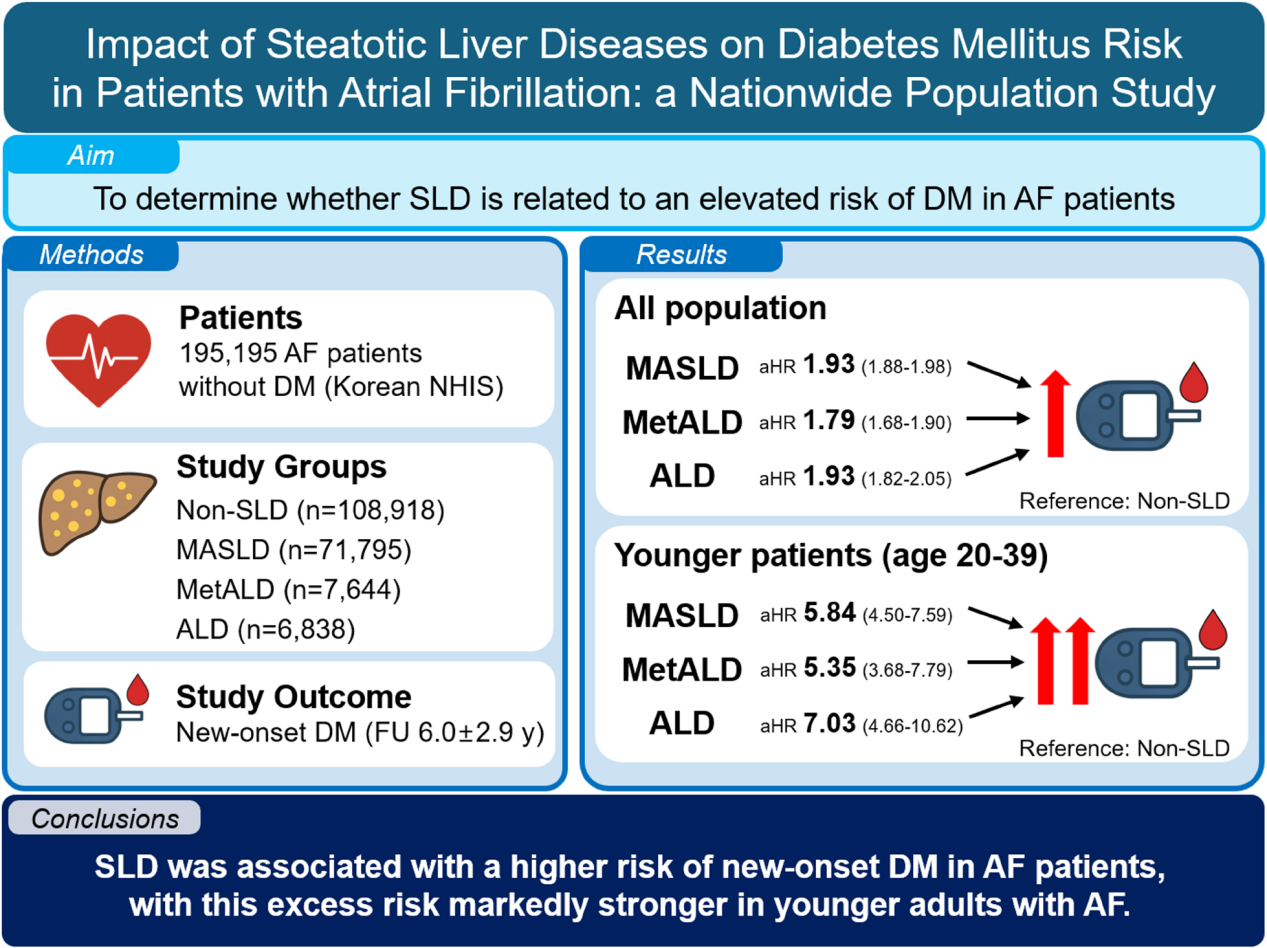

**Supplementary Information:**

The online version contains supplementary material available at 10.1186/s12933-025-02795-5.

## Research insights

**What is currently known about this topic?**

- Coexisting AF and DM increase risks of poor outcomes, such as mortality and ischemic stroke.
- In the general population, SLD is a recognized risk factor of incident DM.

**What is the key research question?**

- Is SLD associated with an increased risk of incident DM in patients with AF?

**What is new?**

- In AF patients, SLD was related to ~2‑fold higher risk of incident DM, compared to non-SLD group.
- This association was stronger in AF patients aged 20-39 years, with DM risk reaching ~5‑ to 7‑fold.

**How might this study influence clinical practice?**

- Proactive DM prevention in AF patients with SLD might be considered, particularly in younger adults.

## Introduction

Atrial fibrillation (AF) is the most common arrhythmic disease in general population, with its prevalence and overall burden continually increasing, reaching more than 59 million cases worldwide in 2019 [1]. AF is associated with various adverse outcomes, including all-cause death, cardiovascular death, and ischemic stroke [2, 3].

Approximately 30% of patients with AF concurrently have diabetes mellitus (DM), which is a major cardiovascular risk factor [4, 5]. Comorbid DM in patients with AF is known to have a worse prognosis, including significantly higher risks of thromboembolism, renal dysfunction, heart failure, all-cause mortality, and cardiovascular mortality [5, 6]. Conversely, AF in patients with DM is also linked to increased risks of all-cause death, cardiovascular death, and other DM-related complications, including macrovascular disease, diabetic nephropathy, and diabetic foot [7, 8]. These synergistic effects of AF and DM are likely multifactorial, driven in part by the cardiac structural and electrical remodeling effects of DM and the pro-thromboembolic effects of AF [5, 8, 9].

Meanwhile, steatotic liver disease (SLD), which encompasses a broad spectrum of diseases characterized by inappropriate hepatic steatosis, is the most common liver disease, with a prevalence of about 30% in the general population [10]. The classification of SLD has been recently updated and now includes hepatic steatosis arising from various causes, such as metabolic dysfunction-associated steatotic liver disease (MASLD), alcohol-related liver disease (ALD), MASLD with increased alcohol intake (MetALD), and other less common etiologies [11]. Multiple studies have shown that SLD increases the risk of DM, likely stemming from the insulin resistance associated with SLD [10, 12].

Given these findings, examining the relationship between SLD and DM in patients with AF is important, although limited data currently exist. Therefore, this nationwide study aimed to investigate the risk of incident DM across different SLD subtypes (MASLD, MetALD, and ALD) compared to those without SLD among non-diabetic patients with AF.

## Methods

### Data source and study population

This retrospective cohort study was conducted with data from the National Health Insurance Service (NHIS) of South Korea, which offers compulsory insurance coverage for all individuals in South Korea. All adults aged ≥ 20 in South Korea are recommended to undergo regular biennial health check-ups fully covered by the NHIS [13]. The NHIS database includes comprehensive individual medical information such as demographic data, anthropometric and biochemical measurements, clinical measurements, medical diagnoses based on the International Classification of Diseases 10th Revision (ICD-10), treatment and medication records, as well as lifestyle questionnaires [13, 14]. Using this database, we examined how MASLD, MetALD, and ALD were related to the incidence of DM in patients with AF.

**Fig. 1:**
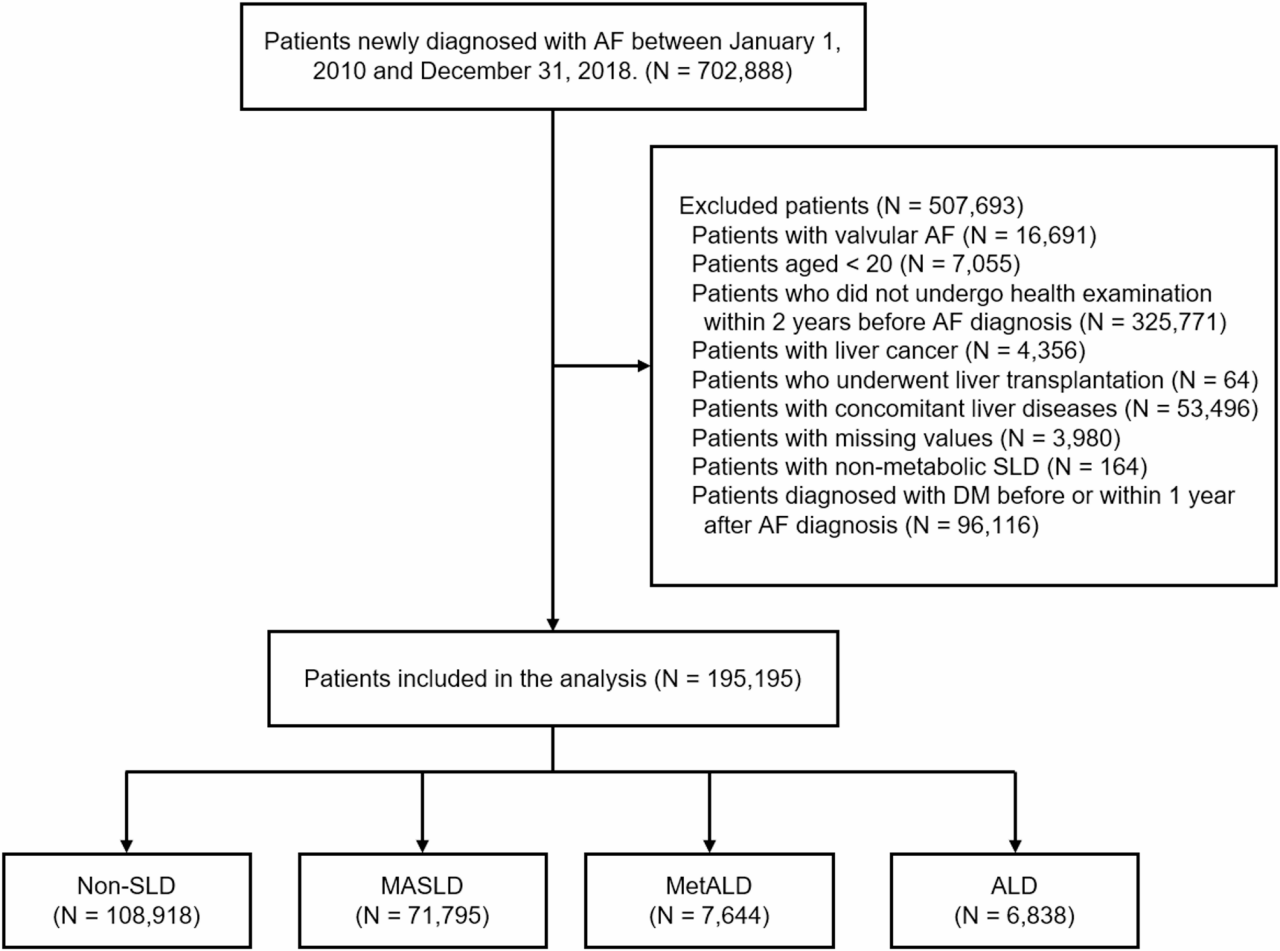
Study flow

This study initially included 702,888 patients who developed AF between January 1, 2010 and December 31, 2018. We excluded patients under the age of 20, those with missing data, and those who did not undergo a regular health check-ups within 2 years before their AF diagnosis. We also excluded those with valvular AF (defined as AF with moderate to severe mitral stenosis or mechanical valves), those with liver cancer or concomitant liver diseases, those who underwent liver transplantation, those with non-metabolic SLD (described below), and those who were diagnosed with DM before or within 1 year after their AF diagnosis. These exclusions resulted in a final study population of 195,195 patients (Fig. 1). Detailed definitions of inclusion and exclusion criteria are summarized in Supplementary Table 1.

This study was approved by the Institutional Review Board of Seoul National University Hospital (IRB no. E-2412-101-1597), and was performed in accordance with the World Medical Association Declaration of Helsinki. The requirement for informed consent was waived as all personally identifiable information was inaccessible.

### Definitions of MASLD, MetALD and ALD

The standard methods for diagnosing SLD include histological or imaging approaches, which are invasive or expensive and thus unsuitable for large-scale screening. Therefore, in this study, we used fatty liver index (FLI) to define SLD. FLI is determined from triglyceride (TG) levels, gamma-glutamyl-transferase (GGT) levels, body mass index (BMI), and waist circumference (WC), hence appropriate for defining hepatic steatosis in large-scale studies [10, 15]. FLI values range from 0 to 100; an FLI < 30 can be employed to rule out hepatic steatosis, and an FLI ≥ 60 can be employed to rule it in [15]. For the criteria of SLD, although both FLI ≥ 30 and FLI ≥ 60 have been used in previous studies [16–18], prior data suggest that FLI ≥ 30 can serve as a cut-off for detecting SLD in the Asian population, yielding a sensitivity of 73–80% and specificity of 72–76% [19, 20]. Therefore, in this study, patients with FLI ≥ 30 were classified as having SLD, and those with FLI < 30 as non-SLD.

Among patients with SLD, cardiometabolic risk factors were assessed to define MASLD, MetALD, and ALD [11]. The cardiometabolic risk factors included the following five components: (1) BMI ≥ 23 kg/ m^2^ or WC ≥ 90 cm (males)/85 cm (females); (2) fasting glucose ≥ 100 mg/dL, type 2 DM, or treatment for type 2 DM; (3) blood pressure ≥ 130/85 mmHg or treatment for hypertension; (4) plasma TG ≥ 150 mg/dL or lipid-lowering treatment; and (5) plasma high-density lipoprotein cholesterol (HDL-C) ≤ 40 mg/dL (males)/≤ 50 mg/dL (females) or lipid-lowering treatment [11]. For the WC criterion, the cut-off values of 90 cm for males and 85 cm for females are widely accepted in South Korea to define abdominal obesity and are considered to reflect the morbidity associated with increasing WC [18, 21]. Patients with SLD but no cardiometabolic risk factors were considered to have non-metabolic SLD and were excluded from the study to ensure homogeneity of study group, as described above.

Patients with SLD and one or more cardiometabolic risk factor were further classified according to their alcohol intake: those consuming < 30 g/day (males)/< 20 g/day (females) were defined as MASLD, those consuming 30–60 g/day (males)/20–50 g/day (females) as MetALD, and those consuming ≥ 60 g/day (males)/≥ 50 g/day (females) as ALD [11, 22]. However, because patients often under-report their alcohol consumption due to stigma, memory issues, or cognitive impairment, and because MASLD patients with alcohol use disorders experience poorer outcomes than those without [10, 23–25], patients with SLD and ≥ 1 cardiometabolic risk factor who had a diagnosis of alcohol or substance abuse/misuse (detailed definitions in Supplementary Table 1) were classified as ALD regardless of their daily alcohol consumption [23]. Consequently, the entire study population was categorized into four groups: non-SLD, MASLD, MetALD, and ALD.

### Covariates

Baseline characteristics including sociodemographic data, anthropometric measurements, self-reported lifestyle behaviors (from questionnaires), biochemical and clinical measurements, comorbidities (hypertension, dyslipidemia and chronic kidney disease), and the use of oral anticoagulants including direct oral anticoagulants and vitamin K antagonists, were collected. Based on the collected information, clinical scores including Charlson Comorbidity Index (CCI) scores [26], CHA_2_DS_2_-VASc scores [27], HAS-BLED scores [28], were calculated. Detailed definitions of comorbidities and clinical scores are summarized in Supplementary Tables 1 and 2.

Low-income status was defined as belonging in the lowest income quartile and receiving public medical assistance. Drinking status was categorized into four groups according to daily alcohol intake: non-drinkers consumed 0 g/day; moderate drinkers consumed more than 0 but less than 30 g/day (males) or 20 g/day (females); heavy drinkers consumed 30–60 g/day (males) or 20–50 g/day (females); and alcoholics consumed ≥ 60 g/day (males) or ≥ 50 g/day (females). Patients were considered to exercise regularly if they engaged in moderate-intensity exercise on more than four days per week or vigorous-intensity exercise on more than two days per week.

### Study outcomes and follow-up

We evaluated the risk of newly diagnosed DM during the follow-up period according to the SLD group. New-onset DM was defined as meeting at least one of the following conditions: (1) an ICD-10 diagnosis code of E11–E14 combined with the use of any hypoglycemic agent, or (2) a fasting blood sugar level ≥ 126 mg/dL. The index date of follow-up was defined as the baseline health examination date, which was conducted within 2 years prior to the diagnosis of AF. The follow-up continued until earliest occurrence of one of the followings: the diagnosis of new-onset DM, death, or end of the follow-up (December 31, 2022).

### Statistical analysis

Patient data were presented as means ± standard deviations for continuous variables, and as numbers (%) for categorical variables. One-way analysis of variance and the chi-square test were employed to compare continuous and categorical variables, respectively, among the SLD groups.

The incidence rates (IRs) of DM were derived by dividing the number of newly diagnosed DM cases by the follow-up duration and were expressed per 1,000 person-years (PY). The risk of DM in each SLD group, compared to the non-SLD group, was expressed as hazard ratios (HRs) with 95% confidence intervals (CIs) estimated with using Cox proportional hazards regression models. Model 1 was unadjusted, Model 2 was adjusted for age and sex, and Model 3 was further adjusted for age, sex, low-income level, smoking status (never, previous, and current smoker), regular exercise, CCI score, hypertension, dyslipidemia, and chronic kidney disease. We validated the proportional hazards assumption using the log-log cumulative survival plots.

Several subgroup analyses were performed according to age strata (20–39, 40–49, 50–59, 60–69, 70–79, and ≥ 80 years), sex, income level, obesity status (BMI ≥ 25 kg/ m^2^), smoking status, physical activity status, comorbidities, and CHA_2_DS_2_-VASc score strata (0, 1, 2, ≥ 3) to assess the impact of these variables on the risk of DM. We assessed the robustness of our results with two sensitivity analyses: (1) applying a stricter SLD definition (FLI ≥ 60), and (2) excluding the patients who developed DM within two years of AF diagnosis.

All statistical analyses were performed using SAS 9.4 software (SAS Institute, Cary, NC). A two-sided p-value < 0.05 was used to reject the null hypothesis.

## Results

### Baseline characteristics

The baseline characteristics of the 195,195 AF patients according to the SLD groups are summarized in Table 1. The mean age was 64.4 ± 13.0 years, and 112,310 (57.5%) were men. A mean follow-up duration was 6.0 ± 2.9 years, and 25,362 (13.0%) patients were newly diagnosed with DM. Among the total population, 108,918 (55.8%) had non-SLD, while 71,795 (36.8%), 7,644 (3.9%), and 6,838 (3.5%) were classified as MASLD, MetALD, and ALD, respectively.

**Table 1:** Baseline characteristics of the study population

	Total	SLD group				*p*-value
		non-SLD	MASLD	MetALD	ALD	
No. of patients	195,195	108,918	71,795	7644	6838	
Age (years)	64.36 ± 12.99	65.24 ± 13.46	64.04 ± 12.21	58.13 ± 11.76	60.60 ± 11.72	< 0.0001
Males	112,310 (57.5)	51,743 (47.5)	47,139 (65.7)	7279 (95.2)	6149 (89.9)	< 0.0001
Low-income status ^a^	40,241 (20.6)	23,009 (21.1)	14,389 (20.0)	1349 (17.7)	1494 (21.9)	< 0.0001
Smoking status						< 0.0001
Never-smoker	119,345 (61.1)	75,236 (69.1)	40,172 (56.0)	1736 (22.7)	2201 (32.2)	
Ex-smoker	42,924 (22.0)	19,357 (17.8)	18,548 (25.8)	2871 (37.6)	2148 (31.4)	
Current smoker	32,926 (16.9)	14,325 (13.2)	13,075 (18.2)	3037 (39.7)	2489 (36.4)	
Alcohol intake ^b^						< 0.0001
Non-drinker	119,607 (61.3)	76,321 (70.1)	41,904 (58.4)	0 (0)	1382 (20.2)	
Moderate drinker	59,980 (30.7)	28,044 (25.8)	29,891 (41.6)	0 (0)	2045 (29.9)	
Heavy drinker	12,321 (6.3)	3730 (3.4)	0 (0)	7644 (100)	947 (13.9)	
Alcoholic	3287 (1.7)	823 (0.8)	0 (0)	0 (0)	2464 (36.0)	
Regular exercise	40,269 (20.6)	22,357 (20.5)	14,629 (20.4)	1770 (23.2)	1513 (22.1)	< 0.0001
Comorbidities						
Hypertension	143,072 (73.3)	74,176 (68.1)	57,500 (80.1)	5923 (77.5)	5473 (80.0)	< 0.0001
Dyslipidemia	88,560 (45.4)	43,414 (39.9)	38,440 (53.5)	3438 (45.0)	3268 (47.8)	< 0.0001
CKD	22,079 (11.3)	11,846 (10.9)	9272 (12.9)	439 (5.7)	522 (7.6)	< 0.0001
Height (cm)	162.00 ± 9.79	160.18 ± 9.59	163.47 ± 9.71	169.23 ± 7.11	167.42 ± 7.82	< 0.0001
Weight (kg)	64.16 ± 11.81	58.18 ± 8.97	71.34 ± 10.43	74.59 ± 10.75	72.39 ± 11.30	< 0.0001
BMI (kg/m^2^)	24.34 ± 3.28	22.62 ± 2.45	26.66 ± 2.83	25.99 ± 2.90	25.76 ± 3.12	< 0.0001
Waist circumference (cm)	83.71 ± 8.95	78.69 ± 6.90	90.16 ± 6.90	89.75 ± 7.26	89.39 ± 7.62	< 0.0001
Fasting glucose (mg/dL)	96.09 ± 11.39	94.41 ± 10.99	97.91 ± 11.44	99.91 ± 11.76	99.44 ± 12.01	< 0.0001
Systolic BP (mmHg)	127.15 ± 15.89	125.14 ± 15.92	129.65 ± 15.50	130.03 ± 15.29	129.72 ± 15.45	< 0.0001
Diastolic BP (mmHg)	78.04 ± 10.48	76.47 ± 10.21	79.79 ± 10.40	81.55 ± 10.79	80.79 ± 10.69	< 0.0001
Total cholesterol (mg/dL)	191.51 ± 38.15	187.51 ± 36.88	196.99 ± 39.19	196.30 ± 37.49	192.37 ± 39.78	< 0.0001
HDL-C (mg/dL)	54.01 ± 16.96	56.36 ± 16.70	50.42 ± 16.72	54.12 ± 17.50	54.14 ± 16.29	< 0.0001
LDL-C (mg/dL)	112.14 ± 35.06	111.61 ± 33.75	114.21 ± 36.46	107.63 ± 35.46	103.82 ± 37.82	< 0.0001
eGFR (mL/min/1.73m^2^) ^c^	84.41 ± 42.49	85.17 ± 40.06	82.51 ± 44.61	87.98 ± 47.47	88.03 ± 49.98	< 0.0001
TG (mg/dL) ^d^	112.06 (111.80-112.31)	89.20 (88.97–89.42)	148.17 (147.68-148.67)	157.26 (155.49-159.05)	154.66 (152.76-156.58)	< 0.0001
AST (IU/L) ^d^	24.95 (24.91–24.98)	23.45 (23.41–23.49)	26.16 (26.09–26.23)	29.64 (29.39–29.90)	33.45 (33.04–33.86)	< 0.0001
ALT (IU/L) ^d^	20.94 (20.90-20.99)	18.04 (17.99–18.08)	24.75 (24.66–24.84)	27.32 (27.03–27.62)	29.04 (28.66–29.43)	< 0.0001
GGT (IU/L) ^d^	28.99 (28.89–29.08)	21.06 (21.00-21.13)	38.76 (38.56–38.95)	71.79 (70.66–72.94)	80.74 (79.04–82.47)	< 0.0001
Medication use						
OAC ^e^	109,313 (56.0)	57,463 (52.8)	43,492 (60.6)	4563 (59.7)	3795 (55.5)	< 0.0001
CHA_2_DS_2_-VASc score	2.63 ± 1.69	2.74 ± 1.71	2.60 ± 1.68	1.80 ± 1.37	2.07 ± 1.49	< 0.0001
HAS-BLED score	2.88 ± 1.18	2.84 ± 1.21	2.94 ± 1.16	2.61 ± 1.09	3.08 ± 1.12	< 0.0001
CCI score						< 0.0001
0	19,397 (9.9)	10,796 (9.9)	7094 (9.9)	1049 (13.7)	458 (6.7)	
1	32,454 (16.6)	17,869 (16.4)	12,106 (16.9)	1546 (20.2)	933 (13.6)	
≥ 2	143,344 (73.4)	80,253 (73.7)	52,595 (73.3)	5049 (66.1)	5447 (79.7)	

Values are expressed as mean ± SD or number (percentage) unless otherwise stated

*SLD* steatotic liver disease; *MASLD* metabolic dysfunction-associated steatotic liver disease; *MetALD* metabolic dysfunction-associated steatotic liver disease with increased alcohol intake; *ALD* alcohol-associated liver disease; *CKD* chronic kidney disease; *BMI* body mass index; *BP* blood pressure; *HDL-C* high-density lipoprotein cholesterol; *LDL-C* low-density lipoprotein cholesterol; *eGFR* estimated glomerular filtration rate; *AST* aspartate aminotransferase; *ALT* alanine aminotransferase; *GGT* gamma-glutamyl-transferase; *OAC* oral anticoagulant; *CCI* Charlson comorbidity index; *CI* confidence interval

^a^Defined as lowest income quartile and receiving public medical aid

^b^Categorized based on daily alcohol consumption - moderate drinkers: >0 and < 30 g/day (males) or < 20 g/day (females); heavy drinkers: 30–60 g/day (males) or 20–50 g/day (females); and alcoholics: ≥60 g/day (males) or ≥ 50 g/day (females)

^c^Calculated with Modification of Diet in Renal Disease (MDRD) equation; eGFR = 186.3×(Serum creatinine)^-1.154^×(Age)^-0.203^ × (0.742 if female)

^d^Expressed as geometric mean (95% CI)

^e^Including vitamin K antagonists and direct oral anticoagulants

A higher percentage of males were present in the MetALD and ALD groups than in the non-SLD and MASLD groups. Patients were older in the non-SLD and MASLD groups than in the MetALD and ALD groups. Overall, patients with SLD were metabolically less healthy than those without SLD. The proportions of patients with hypertension and dyslipidemia were lowest in the non-SLD group (68.1% and 39.9%, respectively), as was the mean BMI (22.6 ± 2.5 kg/m^2^). Mean levels of fasting glucose, total cholesterol, TG, aspartate aminotransferase (AST), alanine aminotransferase (ALT), and GGT were lowest in the non-SLD group.

The median interval between the AF diagnosis and the last health check-up before the AF diagnosis was 0.90 years (Interquartile range 0.40–1.48 years), and 107,136 patients (54.9% of the final cohort) had their health check-up within one year before the AF diagnosis.

### Risk of newly diagnosed DM across different SLD groups in patients with AF

**Fig. 2:**
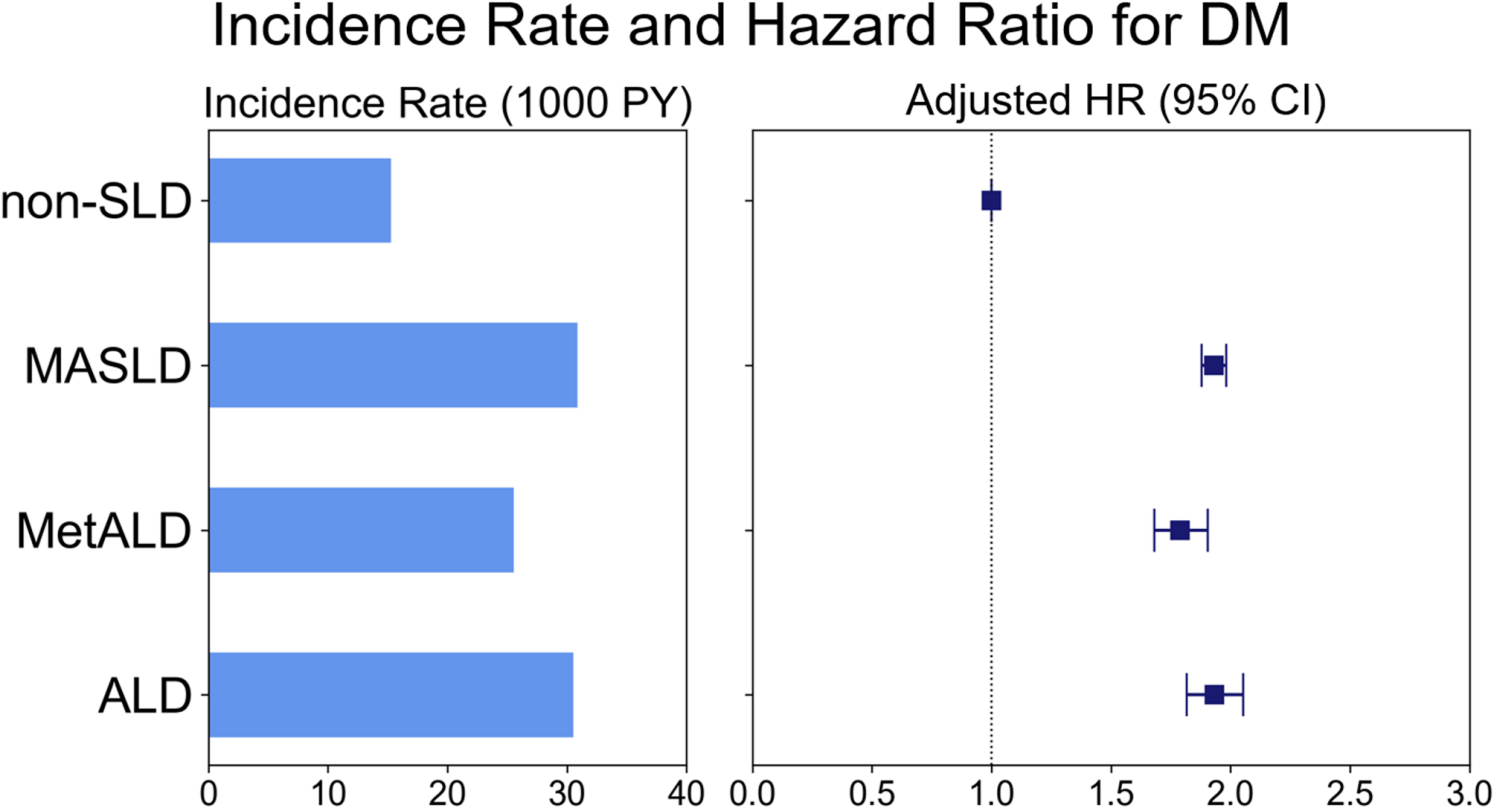
Incidence rate and hazard ratio for DM across different SLD groups in patients with AF

The number of patients with newly diagnosed DM was 10,013 (9.2%) in the non-SLD group, 12,939 (18.0%) in the MASLD group, 1,195 (15.6%) in the MetALD group, and 1,215 (17.8%) in the ALD group. The IRs of newly diagnosed DM per 1,000 PY were 15.19 in the non-SLD group and 30.81, 25.48, and 30.46 in the MASLD, MetALD, and ALD groups, respectively (Table 2; Fig. 2).

**Table 2:** The risk of incident DM across different SLD groups in patients with AF

SLD group	DM cases	Follow-up duration (PY)	IR (1000 PY)	HR (95% CI)		
				Model 1	Model 2	Model 3
Non-SLD	10,013	659275.22	15.19	1 (Ref.)	1(Ref.)	1 (Ref.)
MASLD	12,939	420025.50	30.81	2.034 (1.981–2.087)	2.098 (2.043–2.154)	1.930 (1.879–1.983)
MetALD	1195	46906.94	25.48	1.679 (1.582–1.783)	2.010 (1.890–2.138)	1.789 (1.682–1.904)
ALD	1215	39887.91	30.46	2.009 (1.893–2.133)	2.271 (2.137–2.413)	1.932 (1.817–2.054)

*IR* incidence rate; *PY* person-years; *HR* hazard ratio; *CI* confidence interval; *MASLD* metabolic dysfunction-associated steatotic liver disease; *MetALD* metabolic dysfunction-associated steatotic liver disease with increased alcohol intake; *ALD* alcohol-associated liver disease; *CCI* Charlson comorbidity index

Model 1 was unadjusted

Model 2 was adjusted for age and sex

Model 3 was further adjusted for low-income level, smoking status, regular exercise, CCI score, hypertension, dyslipidemia and chronic kidney disease

Compared to the non-SLD group, all three SLD groups showed an increased risk of incident DM in all models, while the MetALD group showed a slightly lower risk than the other two SLD groups. After multivariable adjustment (Model 3), the MASLD and ALD groups presented with approximately twice the risk of incident DM compared to the non-SLD group, with adjusted HRs and 95% CIs of 1.930 (1.879–1.983) for the MASLD group, 1.789 (1.682–1.904) for the MetALD group, and 1.932 (1.817–2.054) in the ALD group (Table 2; Fig. 2).

### Risk of newly diagnosed DM across different SLD groups in AF patients according to age strata

**Fig. 3:**
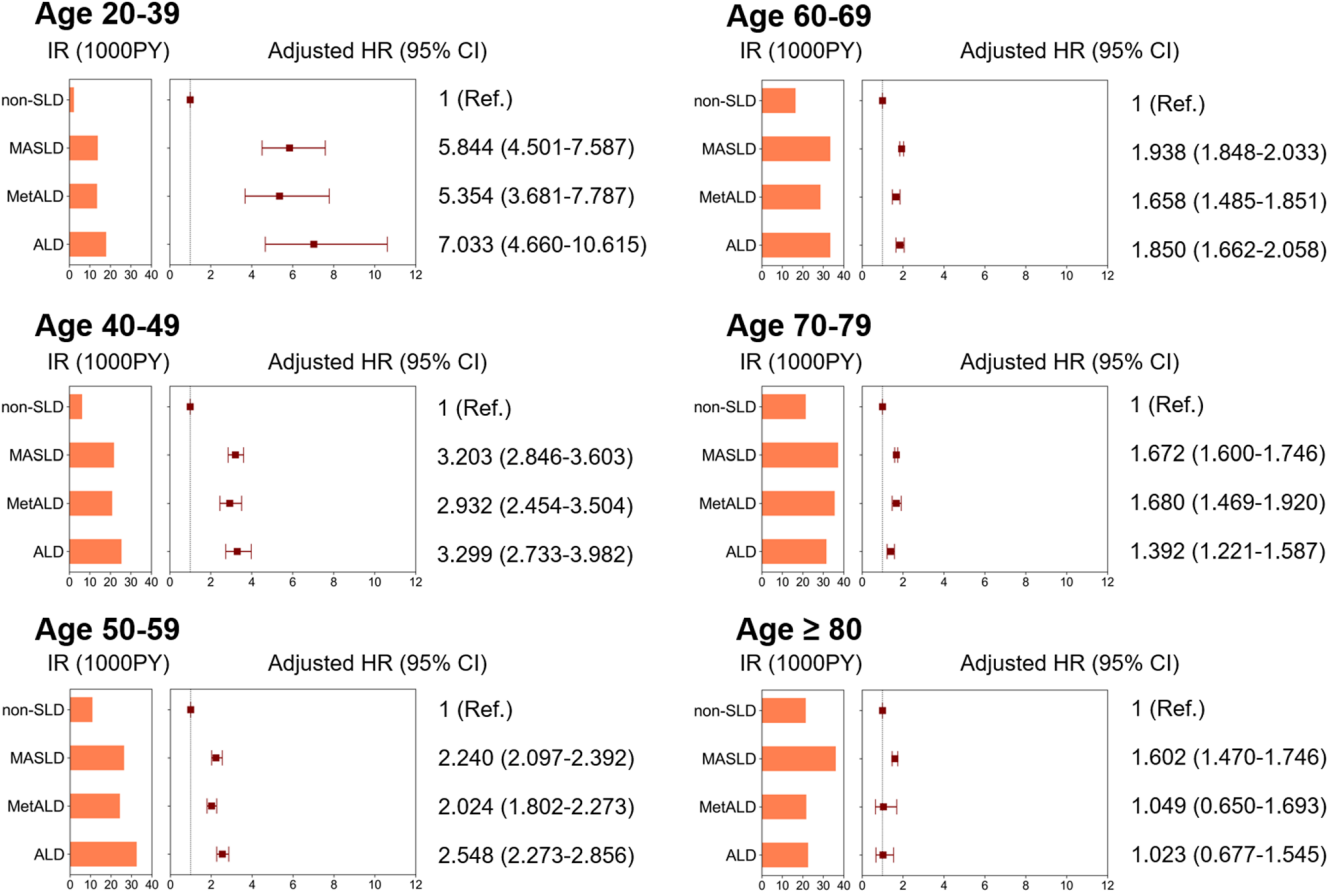
Incidence and hazard ratio for DM across different SLD groups according to age strata in patients with AF

The IRs and HRs with 95% CIs for incident DM in each SLD group by age strata are summarized in Table 3; Fig. 3. We examined how SLDs influence the risk of incident DM in different age strata (20–39, 40–49, 50–59, 60–69, 70–79, and ≥ 80 years). Compared to the non-SLD group, the risks of newly diagnosed DM in SLD groups were significantly accentuated among younger patients, and the adjusted HRs generally decreased as age increased (p for interaction < 0.0001). Specifically, in patients aged 20–39 years, SLD groups showed approximately a 5- to 7-fold higher risk of DM compared to non-SLD, 3-fold in those aged 40–49, and > 2-fold in those aged 50–59. In every age group, the MASLD and ALD groups had slightly higher risks of incident DM than the MetALD group.

**Table 3:** The risk of incident DM across different SLD groups in patients with AF according to age strata

Age	SLD group	No. of patients	DM cases	Follow-up duration (PY)	IR (1000 PY)	aHR (95% CI)
						Model 3
20–39						
	Non-SLD	4918	73	36241.68	2.01	1 (Ref.)
	MASLD	2649	252	18356.26	13.73	5.844 (4.501–7.587)
	MetALD	486	44	3300.06	13.33	5.354 (3.681–7.787)
	ALD	276	33	1855.55	17.78	7.033 (4.660-10.615)
40–49						
	Non-SLD	9411	402	66866.42	6.01	1 (Ref.)
	MASLD	6556	927	42985.74	21.57	3.203 (2.846–3.603)
	MetALD	1296	176	8495.14	20.72	2.932 (2.454–3.504)
	ALD	939	151	5980.91	25.25	3.299 (2.733–3.982)
50–59						
	non-SLD	19,445	1439	133216.53	10.80	1 (Ref.)
	MASLD	14,795	2498	95129.53	26.26	2.240 (2.097–2.392)
	MetALD	2380	365	15077.76	24.21	2.024 (1.802–2.273)
	ALD	1908	381	11770.55	32.37	2.548 (2.273–2.856)
60–69						
	Non-SLD	27,882	2889	177697.73	16.26	1 (Ref.)
	MASLD	21,108	4227	126941.04	33.30	1.938 (1.848–2.033)
	MetALD	2089	363	12739.76	28.49	1.658 (1.485–1.851)
	ALD	1999	388	11651.06	33.30	1.850 (1.662–2.058)
70–79						
	Non-SLD	32,871	3943	185668.50	21.24	1 (Ref.)
	MASLD	20,762	4145	111817.17	37.07	1.672 (1.600-1.746)
	MetALD	1209	230	6505.67	35.35	1.680 (1.469–1.920)
	ALD	1457	239	7604.64	31.43	1.392 (1.221–1.587)
≥ 80						
	Non-SLD	14,391	1267	59584.35	21.26	1 (Ref.)
	MASLD	5925	890	24795.76	35.89	1.602 (1.470–1.746)
	MetALD	184	17	788.54	21.56	1.049 (0.650–1.693)
	ALD	259	23	1025.20	22.43	1.023 (0.677–1.545)
						p-for-interaction < 0.0001

Model 3 was adjusted for age, sex, low-income level, smoking status, regular exercise, CCI score, hypertension, dyslipidemia and chronic kidney disease

*DM* diabetes mellitus; *SLD* steatotic liver disease; *AF* atrial fibrillation; *IR* incidence rate, *PY* person-years; *aHR* adjusted hazard ratio; *CI* confidence interval; *MASLD* metabolic dysfunction-associated steatotic liver disease; *MetALD* metabolic dysfunction-associated steatotic liver disease with increased alcohol intake; *ALD* alcohol-associated liver disease; *CCI* Charlson comorbidity index

In the age 20–39 years group, adjusted HRs (Model 3) with 95% CIs for incident DM compared to non-SLD were 5.844 (4.501–7.587) in MASLD group, 5.354 (3.681–7.787) in MetALD group, and 7.033 (4.660–10.615) in ALD group, while in the age 40–49 years group, the adjusted HRs with 95% CIs were 3.203 (2.846–3.603), 2.932 (2.454–3.504), and 3.299 (2.733–3.982), respectively.

### Subgroup analysis

The results of the subgroup analyses examining the relationship between DM risk and SLDs are presented in Supplementary Table 3. Significant interactions were observed when stratifying patients by sex, presence of hypertension, presence of dyslipidemia, presence of chronic kidney disease, and CHA_2_DS_2_-VASc score. For hypertension, dyslipidemia, and chronic kidney disease, the presence of each comorbidity increased the IRs of incident DM, while the adjusted HRs for incident DM in SLD groups compared to the non-SLD group were accentuated in those without the comorbidity than in those with it. Categorizing the population by income level, presence of obesity, smoking status (never or previous smoker vs. current smoker), and engagement in regular exercise did not show significant interactions.

### Sensitivity analysis

The first sensitivity analysis was performed using SLD criteria of FLI cut-off ≥ 60, including 195,337 patients in the analysis (Supplementary Fig. 1), and the results were consistent with our primary findings. The IRs of newly diagnosed DM per 1,000 PY were 19.03 in the non-SLD group, and 39.39, 32.90, and 38.33 in the MASLD, MetALD, and ALD group, respectively.

Without adjustment, HRs with 95% CIs for newly diagnosed DM compared to the non-SLD group were 2.077 (2.013–2.144) in the MASLD group, 1.730 (1.607–1.863) in the MetALD group, and 2.020 (1.875–2.175) in the ALD group. After multivariable adjustment (Model 3), adjusted HRs with 95% CIs were 2.042 (1.977–2.110), 1.852 (1.718–1.997), and 1.991 (1.846–2.147) in the MASLD, MetALD, and ALD groups, respectively (Supplementary Table 4).

The second sensitivity analysis, excluding patients who developed DM within two years of their AF diagnosis (Supplementary Fig. 2), also produced results concordant with the primary analysis. MASLD, MetALD, and ALD were each associated with approximately 1.7- to 1.9-fold higher risk of incident DM overall (Supplementary Table 5). Among patients aged 20–39 years, adjusted HRs (Model 3) with 95% CIs were 5.893 (4.478–7.756), 5.202 (3.488–7.758), and 6.736 (4.327–10.485) in the MASLD, MetALD, and ALD groups, respectively (Supplementary Table 6).

## Discussion

This study was a large, nationwide investigation with the following principal findings: (1) all three SLD groups were associated with a higher risk of incident DM compared to the non-SLD group in patients with AF; (2) the risk of incident DM in SLD groups compared to non-SLD group was prominently higher in younger patients with AF, and this difference diminished as age increased; and (3) the elevated risk of incident DM in SLD groups, compared to non-SLD, was attenuated in AF patients with comorbidities than in those without.

To the best of our knowledge, this is the first study to investigate the risk of incident DM in patients with both SLD and AF using large population-based data.

Several previous studies and meta-analyses have reported that MASLD is related to approximately 2- to 5-fold risk of newly diagnosed DM among the general population [29–31]. Consistent with previous studies, our findings indicate that MASLD, MetALD, and ALD are associated with a 1.7 to 1.9 times higher risk of incident DM, suggesting a similar risk in patients with SLD and AF to that observed in the general population. The main mechanism by which MASLD contributes to DM development is thought to be increased gluconeogenesis and insulin resistance caused by intrahepatic and extrahepatic fat deposition [32, 33]. This pathophysiology may also involve genetic predisposition. Based on a previous genome-wide association study using Mendelian randomization analysis, at least fifteen core genetic variants have been identified as shared between MASLD and DM, particularly in relation to lipid metabolism and insulin resistance [34].

Another novel aspect of our findings is that MetALD and ALD also contribute to DM development in patients with AF. Although the relationship between MASLD and incident DM has been investigated previously, few studies have assessed MetALD and DM or ALD and DM, especially after the revision of the SLD nomenclature. Traditionally, MASLD and ALD were viewed as distinct disease categories, but recently the synergistic effects of metabolic dysfunction and alcohol intake on SLD have received more attention [10]. Our results align with this updated perspective on SLD, suggesting that SLD groups other than MASLD are also associated with DM development in patients with AF.

Another major finding of this study is that the risk of incident DM in SLD groups, compared to non-SLD, was more pronounced among younger patients with AF than among older ones. This result aligns with a prior nationwide cohort study in Korea, which demonstrated that MASLD patients aged 20–39 years had approximately 6-fold higher risk of new-onset DM compared to those without MASLD [35]. The implications of these findings are especially significant for younger AF patients with SLD, as they may experience an earlier onset of DM and consequently face prolonged exposure to hyperglycemia.

Previous studies have shown that AF patients with a longer duration of DM are more prone to ischemic stroke and have higher all-cause mortality compared to those with shorter DM duration [36, 37]. In the nationwide Danish registries, for example, when compared to non-diabetic patients with AF, patients with AF and a DM duration exceeding 15 years had a 48% higher risk of thromboembolism and a 76% higher risk of mortality, while those with a DM duration of fewer than 5 years exhibited an 11% greater risk of thromboembolism and a 26% higher risk of mortality [36]. Considering the poorer prognosis associated with a longer duration of DM in patients with AF, these findings highlight the importance of strategies to prevent DM in younger individuals who have concurrent SLD and AF.

An additional finding of this study was that, among patients with AF, the relative increase of DM risk in SLD group was attenuated in those with comorbidities than in those without, suggesting that the impact of SLD on increasing DM risk is more critical in those without comorbidities. This may reflect different baseline metabolic profiles, as individuals without both comorbidities and SLD are likely metabolically healthier than those with comorbidities but without SLD. From a DM prevention standpoint in patients with AF, the influence of SLD on DM risk may be more evident in those without comorbidities, highlighting the potential benefits of preventing SLD in this population.

Given that MASLD, MetALD, and ALD each confer a higher risk of incident DM than non-SLD in patients with AF, potential modifications to AF management may be considered. The contemporary guidelines for AF management emphasize the importance of holistic or integrated care management that includes proactive treatment of comorbidities and lifestyle, including glycemic control in patients with concurrent AF and DM [38–40]. Based on our findings, screening for SLD in non-diabetic patients with AF might be beneficial, as MASLD, MetALD, and ALD are significant predictors of developing DM in this population. Moreover, our subgroup analyses suggested that such screening confers particular benefits in younger non-diabetic individuals with AF and that more intensive DM prevention strategies may be necessary for younger patients with concurrent SLD and AF to avoid worse outcomes resulting from longer duration of concurrent AF and DM.

Another clinical implication of our findings is the potential pharmacological prevention of incident DM in patients with concomitant AF and SLD. Recently, numerous clinical trials evaluating the effect of anti-diabetic medications for MASLD have been completed or are ongoing [41, 42]. These therapies have the potential to offer a promising strategy to manage MASLD and prevent DM simultaneously, in non-diabetic patients with AF and MASLD.

### Limitations

Our study has several limitations. First, because the South Korean population predominantly consists of a relatively homogeneous Asian ethnicity, generalizing these findings to other ethnic groups may be challenging, given the reported ethnic differences in the clinical epidemiology of AF and AF-related complications, such as stroke [43, 44]. Both genetic predisposition and lifestyle factors influencing DM development vary widely across different ethnic cohorts. Second, as this study was conducted retrospectively and given its observational nature, shows associations and not causality between SLD and DM in patients with AF. Third, only about 30% of the original AF cohort were included in the final analysis. A substantial number of patients were excluded because they lacked a health screening visit within 2 years before AF diagnosis, developed DM before or within 1 year of the AF diagnosis, or had concomitant liver disease. These exclusions were required to define SLD with FLI, remove prevalent DM, and avoid misclassification, respectively. However, the attrition might have introduced selection bias and could limit the generalizability of our findings. In particular, since regular health check-ups in South Korea are recommended but not mandatory, individuals who underwent health screening within two years before AF diagnosis are likely to be more health-conscious. This may have led to a “healthy user effect,” potentially resulting in an underestimation of the incidence of DM compared to the general population [45]. Fourth, neither imaging nor histological evaluations were employed to define SLD. However, given that the NHIS database is based on routine health check-ups, performing imaging or liver biopsy on the general population would be impractical. Instead, we utilized FLI, a widely accepted scoring system in similar cohort studies, to ensure adequate sensitivity and specificity for defining SLD. We also performed a sensitivity analysis using a higher FLI threshold of ≥ 60, which yielded results comparable to those of our primary analysis. Nevertheless, further study using imaging or histological data is necessary for a more robust conclusion. Fifth, the approach used to define DM in this study differed slightly from standardized diagnostic criteria [46], potentially underestimating the incidence of DM. Since glycated hemoglobin and 2-hour plasma glucose levels following a 75 g oral glucose tolerance test were unavailable in the NHIS database, these measurements could not be utilized. Finally, other potential confounding factors, such as dietary habits, genetic variation, and family history, were not accounted for, as they were not included in the NHIS data.

## Conclusions

This large, nationwide population-based study demonstrated that among AF patients, those with MASLD, MetALD, or ALD have approximately double the risk of developing DM compared to those without SLD, and this risk is even more pronounced in younger AF patients. Considering the increased complication rates associated with concurrent AF and DM, implementing management strategies to prevent DM in AF patients with SLD could potentially mitigate the risk of DM and its potential impact on AF-related outcomes.

## Electronic supplementary material

Below is the link to the electronic supplementary material.

Supplementary Material 1.

## Data Availability

The datasets used during the current study are available from the corresponding author on reasonable request.

## Abbreviations

AF: Atrial fibrillation
DM: Diabetes mellitus
SLD: Steatotic liver disease
MASLD: Metabolic dysfunction-associated steatotic liver disease
MetALD: MASLD with increased alcohol intake
ALD: Alcohol-related liver disease
NHIS: National health insurance service
ICD: International classification of diseases
FLI: Fatty liver index
BMI: Body mass index
WC: Waist circumference
TG: Triglyceride
GGT: Gamma-glutamyl-transferase
AST: Aspartate aminotransferase
ALT: Alanine aminotransferase
CCI: Charlson comorbidity index
