# Supplementary material for: Impact of steatotic liver diseases on diabetes mellitus risk in patients with atrial fibrillation: a nationwide population study
Source: Cardiovasc Diabetol. 2025 Jun 7;24:242. doi: 10.1186/s12933-025-02795-5 (PMC12144779; doi:10.1186/s12933-025-02795-5)
Supplement: Supplementary file 1 — Supplementary Material 1. [file 12933_2025_2795_MOESM1_ESM.docx]

**Title**

**Impact of steatotic liver diseases on diabetes mellitus risk in patients with atrial fibrillation: a nationwide population study**

**Supplementary Materials**

**Supplementary Table 1.** Definitions of diagnoses and clinical scores.

**Supplementary Table 2.** Definition of Charlson comorbidity index.

**Supplementary Table 3.** Subgroup analyses of the risk of incident DM across different SLD groups in patients with AF.

**Supplementary Table 4.** Sensitivity analysis: the risk of incident DM across different SLD groups in patients with AF using SLD definition of FLI ≥60.

**Supplementary Table 5.** Sensitivity analysis: the risk of incident DM across different SLD groups in patients with AF applying a 2-year lag for DM onset.

**Supplementary Table 6.** Sensitivity analysis: the risk of incident DM across different SLD groups in patients with AF according to age strata applying a 2-year lag for DM onset.

**Supplementary Figure 1.** Study flow for sensitivity analysis using SLD definition of FLI ≥60.

**Supplementary Figure 2.** Study flow for sensitivity analysis applying a 2-year lag for DM onset.

**Supplementary Table 1.** Definitions of diagnoses and clinical scores.

| **Diagnosis** | **ICD-10-CM code and definition** | **Diagnostic definition** |
| --- | --- | --- |
| **Inclusion/exclusion criteria** |  |  |
| **Atrial fibrillation** | I48.0-48.4, I48.9 | Admission ≥ 1 or outpatient department ≥2 |
| **Valvular atrial fibrillation** | I05.0, I05.2, I05.9, Z95.2-Z95.4 | Admission ≥ 1 or outpatient department ≥1 |
| **Diabetes mellitus** | E11-E14; and minimum one prescription of anti-diabetic drugs (sulfonylureas, metformin, meglitinides, thiazolidinediones, dipeptidyl peptidase-4 inhibitors, α-glucosidase inhibitors, or insulin). | Admission ≥1 or outpatient department ≥1 |
|  | *OR* fasting glucose level ≥ 126 mg/dL | Index health examination |
| **Malignant neoplasm of liver and intrahepatic bile ducts** | C22 | Admission ≥1 or outpatient department ≥1 |
| **Liver transplant status** | Z94.4 | Admission ≥1 or outpatient department ≥1 |
| **Concomitant liver disease** |  | Admission ≥1 or outpatient department ≥1 |
| **Drug-induce liver injury** | K71 |  |
| **Viral hepatitis** | B00.81, B15-B19, B25.1 |  |
| **Budd-Chiari syndrome** | I82.0 |  |
| **Liver abscess** | K75.0, A06.4 |  |
| **Hemochromatosis** | E83.1 |  |
| **Wilson’s disease** | E83.0 |  |
| **α1-antitrypsin deficiency** | E88.01 |  |
| **Autoimmune hepatitis** | K75.4 |  |
| **Primary / Secondary biliary cholangitis** | K74.3-K74.4 |  |
| **Other diseases of biliary tract** | K83 |  |
| **Glycogen storage disease** | E74 |  |
| **Related to abuse/misuse of alcohol or other substances** |  |  |
| **Alcohol-induced pseudo-Cushing's syndrome** | E24.4 | Admission ≥1 or outpatient department ≥1 |
| **Alcohol related disorders** | F10 | Admission ≥1 or outpatient department ≥1 |
| **Disorders related to substances other than alcohol** | F11-19 | Admission ≥1 or outpatient department ≥1 |
| **Degeneration of nervous system due to alcohol** | G31.2 | Admission ≥1 or outpatient department ≥1 |
| **Alcoholic polyneuropathy** | G62.1 | Admission ≥1 or outpatient department ≥1 |
| **Alcoholic myopathy** | G72.1 | Admission ≥1 or outpatient department ≥1 |
| **Alcoholic cardiomyopathy** | I42.6 | Admission ≥1 or outpatient department ≥1 |
| **Alcoholic gastritis** | K29.2 | Admission ≥1 or outpatient department ≥1 |
| **Alcoholic liver disease** | K70 | Admission ≥1 or outpatient department ≥1 |
| **Alcohol-induced chronic pancreatitis** | K86.0 | Admission ≥1 or outpatient department ≥1 |
| **Finding of alcohol in blood** | R78.0, Y90 | Admission ≥1 or outpatient department ≥1 |
| **Toxic effect of alcohol** | T51.0, T51.8-T51.9 | Admission ≥1 or outpatient department ≥1 |
| **Alcohol abuse counseling and surveillance** | Z71.4 | Admission ≥1 or outpatient department ≥1 |
| **Comorbidity** |  |  |
| **Chronic kidney disease** | eGFR ^a^ <60 ml/min/1.73m^2^ | Index health examination |
| **Dyslipidemia** | E78; and minimum one prescription of lipid-lowering medication. | Admission or outpatient department ≥1 |
|  | *OR* Total cholesterol ≥ 240 mg/dL | Index health examination |
| **Hypertension** | I10-I13, I15; and minimum one prescription of anti-hypertensive medication (thiazide, loop diuretics, aldosterone antagonist, alpha-/beta-blocker, calcium-channel blocker, angiotensin-converting enzyme inhibitor, or angiotensin II receptor blocker). | Admission≥1 or outpatient department ≥2 |
|  | *OR* systolic/diastolic blood pressure ≥ 140/90 mmHg | Index health examination |
| **Clinical score** | **Definition** | |
| **CHA_2_DS_2_-VASc** | Heart failure (1 point), hypertension (1 point), age (1 point for ≥65 and 2 points for ≥75), diabetes (1 point), previous stroke/systemic embolism/transient ischemic attack (2 points), vascular disease (prior MI or PAD, 1 point) and female sex (1 point). | |
| **HAS-BLED** | Hypertension (1 point), abnormal renal function (1 point), abnormal liver function (1 point), previous stroke (1 point), prior major bleeding or predisposition to bleeding (1 point), labile INR (1 point), age >65 (1 point), usage of antiplatelet agent or NSAID (1 point), alcohol excess (1 point). | |
| **Charlson comorbidity index** | See Supplementary Table 2. | |

^a^ calculated with Modiﬁcation of Diet in Renal Disease (MDRD) equation; eGFR=186.3×(Serum creatinine)^-1.154^×(Age)^-0.203^×(0.742 if female)

Abbreviations: ICD international classification of disease; CM clinical modification; eGFR estimated glomerular filtration rate; MI myocardial infarction, PAD peripheral artery disease; INR international normalized ratio; NSAID non-steroidal anti-inflammatory drug.

**Supplementary Table 2.** Definition of Charlson comorbidity index.

| **Category** | **Weights** | **Disease** | **ICD-10-CM code** |
| --- | --- | --- | --- |
| **Myocardial infarction** | 1 | Acute myocardial infarction | I21 |
|  |  | Subsequent myocardial infarction | I22 |
| **Congestive heart failure** | 1 | Heart Failure | I50 |
| **Peripheral vascular disease** | 1 | Atherosclerosis | I70 |
|  |  | Other peripheral vascular disease | I73 |
| **Cerebrovascular disease** | 1 | Transient cerebral ischemic attacks and related syndromes | G45 |
|  |  | Vascular syndromes of brain in cerebrovascular diseases | G46 |
|  |  | Retinal vascular occlusion | H34 |
|  |  | Cerebrovascular disease | I60-I69 |
| **Dementia** | 1 | Dementia in Alzheimer disease | F00 |
|  |  | Vascular dementia | F01 |
|  |  | Dementia in other disease classified elsewhere | F02 |
|  |  | Unspecified dementia | F03 |
| **Chronic pulmonary disease** | 1 | Chronic lower respiratory diseases | J40-J47 |
|  |  | Lung disease due to external agents | J60-J67 |
| **Rheumatic disease** | 1 | Rheumatoid arthritis with rheumatoid factor | M05 |
| **(connective tissue disorder)** |  | Felty's syndrome | M05.0 |
|  |  | Rheumatoid lung disease with rheumatoid arthritis | M05.1 |
|  |  | Rheumatoid vasculitis with rheumatoid arthritis | M05.2 |
|  |  | Rheumatoid heart disease with rheumatoid arthritis | M05.3 |
|  |  | Rheumatoid myopathy with rheumatoid arthritis | M05.4 |
|  |  | Rheumatoid polyneuropathy with rheumatoid arthritis | M05.5 |
|  |  | Rheumatoid arthritis with involvement of other organs and systems | M05.6 |
|  |  | Rheumatoid arthritis with rheumatoid factor without organ or systems involvement | M05.7 |
|  |  | Other rheumatoid arthritis with rheumatoid factor | M05.8 |
|  |  | Rheumatoid arthritis without rheumatoid factor | M05.9 |
|  |  | Adult-onset Still's disease | M06.1 |
|  |  | Rheumatoid bursitis | M06.2 |
|  |  | Rheumatoid nodule | M06.3 |
|  |  | Inflammatory polyarthropathy | M06.4 |
|  |  | Other specified rheumatoid arthritis | M06.8 |
|  |  | Rheumatoid arthritis, unspecified | M06.9 |
|  |  | Giant cell arteritis with polymyalgia rheumatica | M31.5 |
|  |  | Systemic lupus erythematosus (SLE) | M32 |
|  |  | Drug-induced SLE | M32.0 |
|  |  | SLE with organ or system involvement | M32.1 |
|  |  | Other forms of SLE | M32.8 |
|  |  | SLE, unspecified | M32.9 |
|  |  | Dermatopolymyositis | M33 |
|  |  | Juvenile dermatomyositis | M33.0 |
|  |  | Other dermatomyositis | M33.1 |
|  |  | Polymyositis | M33.2 |
|  |  | Dermatopolymyositis, unspecified | M33.9 |
|  |  | Systemic sclerosis [scleroderma] | M34 |
|  |  | Progressive systemic sclerosis | M34.0 |
|  |  | CR(E)ST syndrome | M34.1 |
|  |  | Systemic sclerosis induced by drug and chemical | M34.2 |
|  |  | Other forms of systemic sclerosis | M34.8 |
|  |  | Systemic sclerosis, unspecified | M34.9 |
|  |  | Other overlap syndromes | M35.1 |
|  |  | Polymyalgia rheumatica | M35.3 |
|  |  | Dermato(poly)myositis in neoplastic disease | M36.0 |
| **Peptic ulcer disease** | 1 | Gastric ulcer | K25 |
|  |  | Duodenal ulcer | K26 |
|  |  | Peptic ulcer, site unspecified | K27 |
|  |  | Gastrojejunal ulcer | K28 |
| **Mild liver disease** | 1 | Chronic viral hepatitis | B18 |
|  |  | Alcoholic fatty liver | K70.0- K70.3, K70.9 |
|  |  | Alcoholic hepatitis |  |
|  |  | Alcoholic fibrosis and sclerosis of liver |  |
|  |  | Alcoholic cirrhosis of liver |  |
|  |  | Alcoholic liver disease, unspecified |  |
|  |  | Toxic liver disease with chronic persistent hepatitis | K71.3- K71.5, K71.7 |
|  |  | Toxic liver disease with chronic lobular hepatitis |  |
|  |  | Toxic liver disease with chronic active hepatitis |  |
|  |  | Toxic liver disease with fibrosis and cirrhosis of liver |  |
|  |  | Chronic hepatitis, not elsewhere classified | K73 |
|  |  | Fibrosis and cirrhosis of liver | K74 |
|  |  | Fatty (change of) liver, not elsewhere classified | K76.0-K76.4, K76.8, K76.9 |
|  |  | Nonalcoholic fatty liver disease |  |
|  |  | Central hemorrhagic necrosis of liver |  |
|  |  | Infarction of liver |  |
|  |  | Hepatic angiomatosis |  |
|  |  | Other specified disease of liver |  |
|  |  | Simple cyst of liver |  |
|  |  | Focal nodular hyperplasia of liver |  |
|  |  | Hepatoptosis |  |
|  |  | Liver disease, unspecified |  |
|  |  | Liver transplant status | Z94.4 |
| **Diabetes without chronic complication** | 1 | with coma | E10.0, 10.1, 10.6, 10.8, 10.9 |
|  |  | with ketoacidosis | E11.0, 11.1, 11.6, 11.8, 11.9 |
|  |  | with other specified complications | E12.0, 12.1, 12.6, 12.8, 12.9 |
|  |  | with unspecified complications | E13.0, 13.1, 13.6, 13.8, 13.9 |
|  |  | without complications | E14.0, 14.1, 14.6, 14.8, 14.9 |
| **Diabetes with chronic complication** | 2 | with renal complications | E10.2, 10.3, 10.4, 10.5, 10.7 |
|  |  | with ophthalmic complications | E11.2, 11.3, 11.4, 11.5, 11.7 |
|  |  | with neurologic complications | E12.2, 12.3, 12.4, 12.5, 12.7 |
|  |  | with peripheral circulatory complications | E13.2, 13.3, 13.4, 13.5, 13.7 |
|  |  | with multiple complications | E14.2, 14.3, 14.4, 14.5, 14.7 |
| **Hemi/paraplegia** | 2 | Tropical spastic paraplegia | G04.1 |
|  |  | Hereditary spastic paraplegia | G11.4 |
|  |  | Spastic quadriplegic cerebral palsy | G80.0 |
|  |  | Spastic diplegic cerebral palsy | G80.1 |
|  |  | Spastic hemiplegic cerebral palsy | G80.2 |
|  |  | Flaccid hemiplegia | G81.0 |
|  |  | Spastic hemiplegia | G81.1 |
|  |  | Hemiplegia, unspecified | G81.9 |
|  |  | Flaccid paraplegia | G82.0 |
|  |  | Spastic paraplegia | G82.1 |
|  |  | Paraplegia, unspecified | G82.2 |
|  |  | Flaccid tetraplegia | G82.3 |
|  |  | Spastic tetraplegia | G82.4 |
|  |  | Tetraplegia, unspecified | G82.5 |
|  |  | Diplegia of upper limbs | G83.0 |
|  |  | Paralytic syndrome, unspecified | G83.9 |
| **Renal disease** | 2 | Hypertensive renal disease | I12 |
|  |  | Hypertensive heart and renal disease with renal failure | I13.1 |
|  |  | Chronic nephritic syndrome | N03 |
|  |  | Unspecified nephritic syndrome | N05 |
|  |  | Chronic kidney disease | N18 |
|  |  | Unspecified kidney failure | N19 |
|  |  | Disorders resulting from impaired renal tubular function | N25 |
|  |  | Care involving dialysis | Z49 |
|  |  | Transplanted organ and tissue status - kidney | Z94.0 |
|  |  | Dependence on renal dialysis | Z99.2 |
| **Cancer** | 2 | Any tumor, malignant neoplasm | C00-76, C97 |
|  |  | Any tumor, in situ neoplasm | D00-09 |
|  |  | Any tumor, Benign neoplasm | D10-36 |
|  |  | Any tumor, Neoplasm of unknown behavior | D37-48 |
|  |  | Leukemia | C91-95 |
|  |  | Lymphoma | C81-86 |
| **Metastatic cancer** | 3 | Metastatic solid tumor | C77-80 |
| **Moderate to severe** | 3 | Esophageal varices | I85 |
| **liver disease** |  | Gastric varices | I86.4 |
|  |  | Esophageal varices without bleeding in diseases classified elsewhere | I98.2 |
|  |  | Alcoholic hepatic failure | K70.4 |
|  |  | Toxic liver disease with hepatic necrosis | K71.1 |
|  |  | Hepatic failure (acute/chronic) due to drugs |  |
|  |  | Chronic hepatic failure | K72.1, K72.9 |
|  |  | Hepatic failure, unspecified |  |
|  |  | Hepatic veno-occlusive disease | K76.5-K76.7 |
|  |  | Portal hypertension |  |
|  |  | Hepatorenal syndrome |  |
| **Acquired immune deficiency syndrome (AIDS)** | 6 | HIV disease resulting in infectious and parasitic diseases | B20 |
|  |  | HIV disease resulting in malignant neoplasm | B21 |
|  |  | HIV disease resulting in other specified diseases | B22 |
|  |  | HIV disease resulting in other conditions | B23 |

Abbreviations: ICD international classification of disease; CM clinical modification; HIV Human immunodeficiency virus.

**Supplementary Table 3.** Subgroup analyses of the risk of incident DM across different SLD groups in patients with AF.

| Subgroup | SLD group | No. of patients | DM cases | Follow-up duration (PY) | IR  (1000 PY) | aHR (95% CI) |
| --- | --- | --- | --- | --- | --- | --- |
|  |  |  |  |  |  | **Model 3** |
| Male |  |  |  |  |  |  |
|  | **non-SLD** | 51,743 | 4,610 | 306251.69 | 15.05 | 1 (Ref.) |
|  | **MASLD** | 47,139 | 7,830 | 278996.52 | 28.06 | 1.833 (1.767-1.902) |
|  | **MetALD** | 7,279 | 1,143 | 44656.37 | 25.60 | 1.747 (1.636-1.866) |
|  | **ALD** | 6,149 | 1,092 | 36008.10 | 30.33 | 1.896 (1.774-2.027) |
| Female |  |  |  |  |  |  |
|  | **non-SLD** | 57,175 | 5,403 | 353023.53 | 15.30 | 1 (Ref.) |
|  | **MASLD** | 24,656 | 5,109 | 141028.98 | 36.23 | 2.044 (1.967-2.125) |
|  | **MetALD** | 365 | 52 | 2250.57 | 23.11 | 1.557 (1.184-2.046) |
|  | **ALD** | 689 | 123 | 3879.82 | 31.70 | 1.783 (1.491-2.132) |
|  |  |  |  |  |  | p-for-interaction =0.0002 |
| No low-income level |  |  |  |  |  |  |
|  | **non-SLD** | 85,909 | 7,761 | 521804.79 | 14.87 | 1 (Ref.) |
|  | **MASLD** | 57,406 | 10,141 | 337532.51 | 30.04 | 1.929 (1.871-1.988) |
|  | **MetALD** | 6,295 | 975 | 38724.96 | 25.18 | 1.818 (1.698-1.947) |
|  | **ALD** | 5,344 | 923 | 31399.39 | 29.40 | 1.922 (1.793-2.061) |
| Low-income level ^a^ |  |  |  |  |  |  |
|  | **non-SLD** | 23,009 | 2,252 | 137470.42 | 16.38 | 1 (Ref.) |
|  | **MASLD** | 14,389 | 2,798 | 82492.99 | 33.92 | 1.936 (1.831-2.047) |
|  | **MetALD** | 1,349 | 220 | 8181.98 | 26.89 | 1.673 (1.455-1.923) |
|  | **ALD** | 1,494 | 292 | 8488.53 | 34.40 | 1.965 (1.738-2.221) |
|  |  |  |  |  |  | p-for-interaction =0.7098 |
| No obesity |  |  |  |  |  |  |
|  | **non-SLD** | 90,775 | 7,881 | 547781.29 | 14.39 | 1 (Ref.) |
|  | **MASLD** | 19,766 | 2,990 | 116749.75 | 25.61 | 1.666 (1.596-1.739) |
|  | **MetALD** | 2,798 | 366 | 17183.13 | 21.30 | 1.467 (1.320-1.631) |
|  | **ALD** | 2,771 | 406 | 16169.53 | 25.11 | 1.588 (1.436-1.757) |
| Obesity ^b^ |  |  |  |  |  |  |
|  | **non-SLD** | 18,143 | 2,132 | 111493.93 | 19.12 | 1 (Ref.) |
|  | **MASLD** | 52,029 | 9,949 | 303275.75 | 32.81 | 1.679 (1.601-1.760) |
|  | **MetALD** | 4,846 | 829 | 29723.81 | 27.89 | 1.626 (1.497-1.765) |
|  | **ALD** | 4,067 | 809 | 23718.38 | 34.11 | 1.789 (1.647-1.943) |
|  |  |  |  |  |  | p-for-interaction =0.1489 |
| Never or ex-smoker |  |  |  |  |  |  |
|  | **non-SLD** | 94,593 | 8,549 | 572668.15 | 14.93 | 1 (Ref.) |
|  | **MASLD** | 58,720 | 10,435 | 341689.78 | 30.54 | 1.938 (1.882-1.996) |
|  | **MetALD** | 4,607 | 676 | 28113.10 | 24.05 | 1.825 (1.684-1.978) |
|  | **ALD** | 4,349 | 718 | 25431.79 | 28.23 | 1.892 (1.751-2.045) |
| Current smoker |  |  |  |  |  |  |
|  | **non-SLD** | 14,325 | 1,464 | 86607.07 | 16.90 | 1 (Ref.) |
|  | **MASLD** | 13,075 | 2,504 | 78335.72 | 31.97 | 1.890 (1.771-2.017) |
|  | **MetALD** | 3,037 | 519 | 18793.84 | 27.62 | 1.729 (1.564-1.912) |
|  | **ALD** | 2,489 | 497 | 14456.12 | 34.38 | 1.975 (1.783-2.187) |
|  |  |  |  |  |  | p-for-interaction =0.5907 |
| No regular exercise |  |  |  |  |  |  |
|  | **non-SLD** | 86,561 | 7,981 | 521296.47 | 15.31 | 1 (Ref.) |
|  | **MASLD** | 57,166 | 10,455 | 332698.24 | 31.42 | 1.961 (1.904-2.021) |
|  | **MetALD** | 5,874 | 931 | 35811.88 | 26.00 | 1.824 (1.700-1.956) |
|  | **ALD** | 5,325 | 951 | 30846.53 | 30.83 | 1.952 (1.822-2.091) |
| Regular exercise ^c^ |  |  |  |  |  |  |
|  | **non-SLD** | 22,357 | 2,032 | 137978.75 | 14.73 | 1 (Ref.) |
|  | **MASLD** | 14,629 | 2,484 | 87327.25 | 28.44 | 1.809 (1.705-1.918) |
|  | **MetALD** | 1,770 | 264 | 11095.06 | 23.79 | 1.669 (1.467-1.898) |
|  | **ALD** | 1,513 | 264 | 9041.38 | 29.20 | 1.857 (1.632-2.112) |
|  |  |  |  |  |  | p-for-interaction =0.0992 |
| No hypertension |  |  |  |  |  |  |
|  | **non-SLD** | 34,742 | 2,063 | 228569.72 | 9.03 | 1 (Ref.) |
|  | **MASLD** | 14,295 | 1,866 | 91060.61 | 20.49 | 2.242 (2.105-2.388) |
|  | **MetALD** | 1,721 | 215 | 11152.67 | 19.28 | 2.242 (1.946-2.582) |
|  | **ALD** | 1,365 | 184 | 8286.21 | 22.21 | 2.367 (2.035-2.754) |
| Hypertension |  |  |  |  |  |  |
|  | **non-SLD** | 74,176 | 7,950 | 430705.50 | 18.46 | 1 (Ref.) |
|  | **MASLD** | 57,500 | 11,073 | 328964.88 | 33.66 | 1.867 (1.813-1.923) |
|  | **MetALD** | 5,923 | 980 | 35754.27 | 27.41 | 1.701 (1.589-1.821) |
|  | **ALD** | 5,473 | 1,031 | 31601.70 | 32.62 | 1.853 (1.734-1.980) |
|  |  |  |  |  |  | p-for-interaction <0.0001 |
| No dyslipidemia |  |  |  |  |  |  |
|  | **non-SLD** | 65,504 | 5,161 | 411460.48 | 12.54 | 1 (Ref.) |
|  | **MASLD** | 33,355 | 5,379 | 203953.63 | 26.37 | 2.049 (1.971-2.130) |
|  | **MetALD** | 4,206 | 568 | 26777.28 | 21.21 | 1.770 (1.621-1.933) |
|  | **ALD** | 3,570 | 574 | 21276.23 | 26.98 | 2.035 (1.865-2.220) |
| Dyslipidemia |  |  |  |  |  |  |
|  | **non-SLD** | 43,414 | 4,852 | 247814.74 | 19.58 | 1 (Ref.) |
|  | **MASLD** | 38,440 | 7,560 | 216071.87 | 34.99 | 1.831 (1.765-1.899) |
|  | **MetALD** | 3,438 | 627 | 20129.66 | 31.15 | 1.800 (1.654-1.960) |
|  | **ALD** | 3,268 | 641 | 18611.68 | 34.44 | 1.836 (1.688-1.996) |
|  |  |  |  |  |  | p-for-interaction =0.0002 |
| No CKD |  |  |  |  |  |  |
|  | **non-SLD** | 97,072 | 8,490 | 594488.99 | 14.28 | 1 (Ref.) |
|  | **MASLD** | 62,523 | 11,019 | 369310.14 | 29.84 | 2.000 (1.943-2.059) |
|  | **MetALD** | 7,205 | 1,096 | 44352.20 | 24.71 | 1.804 (1.691-1.925) |
|  | **ALD** | 6,316 | 1,111 | 36950.57 | 30.07 | 2.007 (1.882-2.140) |
| CKD |  |  |  |  |  |  |
|  | **non-SLD** | 11,846 | 1,523 | 64786.23 | 23.51 | 1 (Ref.) |
|  | **MASLD** | 9,272 | 1,920 | 50715.36 | 37.86 | 1.577 (1.475-1.688) |
|  | **MetALD** | 439 | 99 | 2554.74 | 38.75 | 1.855 (1.513-2.275) |
|  | **ALD** | 522 | 104 | 2937.34 | 35.41 | 1.452 (1.190-1.772) |
|  |  |  |  |  |  | p-for-interaction <0.0001 |
| CHA_2_DS_2_-VASc |  |  |  |  |  |  |
| 0 |  |  |  |  |  |  |
|  | **non-SLD** | 8,428 | 347 | 60534.15 | 5.73 | 1 (Ref.) |
|  | **MASLD** | 6,612 | 696 | 45818.58 | 15.19 | 2.500 (2.198-2.844) |
|  | **MetALD** | 1,184 | 145 | 8203.25 | 17.68 | 2.838 (2.337-3.446) |
|  | **ALD** | 826 | 111 | 5364.96 | 20.69 | 3.065 (2.475-3.797) |
| 1 |  |  |  |  |  |  |
|  | **non-SLD** | 21,347 | 1,292 | 148501.24 | 8.70 | 1 (Ref.) |
|  | **MASLD** | 14,471 | 2,388 | 94211.32 | 25.35 | 2.498 (2.330-2.678) |
|  | **MetALD** | 2,549 | 399 | 16501.72 | 24.18 | 2.275 (2.029-2.550) |
|  | **ALD** | 2,022 | 398 | 12646.88 | 31.47 | 2.820 (2.515-3.162) |
| 2 |  |  |  |  |  |  |
|  | **non-SLD** | 22,795 | 2,028 | 146541.67 | 13.84 | 1 (Ref.) |
|  | **MASLD** | 16,004 | 2,970 | 96469.54 | 30.79 | 2.089 (1.974-2.212) |
|  | **MetALD** | 1,913 | 299 | 11197.18 | 26.70 | 1.781 (1.576-2.013) |
|  | **ALD** | 1,707 | 300 | 9876.33 | 30.38 | 1.941 (1.718-2.193) |
| ≥3 |  |  |  |  |  |  |
|  | **non-SLD** | 56,348 | 6,346 | 303698.16 | 20.90 | 1 (Ref.) |
|  | **MASLD** | 34,708 | 6,885 | 183526.05 | 37.52 | 1.744 (1.684-1.805) |
|  | **MetALD** | 1,998 | 352 | 11004.79 | 31.99 | 1.538 (1.379-1.715) |
|  | **ALD** | 2,283 | 406 | 11999.75 | 33.83 | 1.524 (1.377-1.687) |
|  |  |  |  |  |  | p-for-interaction <0.0001 |

Model 3 was adjusted for age, sex, low-income level, smoking status, regular exercise, CCI score, and hypertension, dyslipidemia and chronic kidney disease.

^a^ Lowest income quartile and receiving public medical aid.

^b^ BMI ≥25.0 kg/m^2^.

^c^ Moderate-intensity exercise ≥5 days/week or vigorous-intensity exercise ≥3 days/week.

Abbreviations: DM diabetes mellitus; SLD steatotic liver disease; AF atrial fibrillation; IR incidence rate, PY person-years; aHR adjusted hazard ratio; CI confidence interval; MASLD metabolic dysfunction-associated steatotic liver disease; MetALD metabolic dysfunction-associated steatotic liver disease with increased alcohol intake; ALD alcohol-associated liver disease; CCI Charlson comorbidity index; BMI body mass index.

**Supplementary Table 4.** Sensitivity analysis: the risk of incident DM across different SLD groups in patients with AF using SLD definition of FLI ≥60.

| SLD group | No. of patients | DM cases | Follow-up duration (PY) | IR (1000 PY) | HR (95% CI) | | |
| --- | --- | --- | --- | --- | --- | --- | --- |
|  |  |  |  |  | **Model 1** | **Model 2** | **Model 3** |
| non-SLD | 166,939 | 19,096 | 1003422.78 | 19.03 | 1 (Ref.) | 1 (Ref.) | 1 (Ref.) |
| MASLD | 21,400 | 4,822 | 122414.13 | 39.39 | 2.077 (2.013-2.144) | 2.266 (2.194-2.340) | 2.042 (1.977-2.110) |
| MetALD | 3,698 | 733 | 22276.29 | 32.90 | 1.730 (1.607-1.863) | 2.101 (1.949-2.264) | 1.852 (1.718-1.997) |
| ALD | 3,300 | 724 | 18887.71 | 38.33 | 2.020 (1.875-2.175) | 2.354 (2.183-2.537) | 1.991 (1.846-2.147) |

Model 1 was unadjusted.

Model 2 was adjusted for age and sex.

Model 3 was adjusted for age, sex, low-income level, smoking status, regular exercise, CCI score, and hypertension, dyslipidemia and chronic kidney disease.

Abbreviations: DM diabetes mellitus; SLD steatotic liver disease; AF atrial fibrillation; IR incidence rate, PY person-years; HR hazard ratio; CI confidence interval; MASLD metabolic dysfunction-associated steatotic liver disease; MetALD metabolic dysfunction-associated steatotic liver disease with increased alcohol intake; ALD alcohol-associated liver disease; CCI Charlson comorbidity index.

**Supplementary Table 5.** Sensitivity analysis: the risk of incident DM across different SLD groups in patients with AF applying a 2-year lag for DM onset.

| SLD group | No. of patients | DM cases | Follow-up duration (PY) | IR (1000 PY) | HR (95% CI) | | |
| --- | --- | --- | --- | --- | --- | --- | --- |
|  |  |  |  |  | **Model 1** | **Model 2** | **Model 3** |
| non-SLD | 104,321 | 8,520 | 552718.78 | 15.41 | 1 (Ref.) | 1 (Ref.) | 1 (Ref.) |
| MASLD | 68,409 | 10,936 | 349941.83 | 31.25 | 2.032 (1.976-2.091) | 2.084 (2.025-2.145) | 1.920 (1.864-1.977) |
| MetALD | 7,370 | 1,015 | 39389.60 | 25.77 | 1.674 (1.569-1.787) | 1.968 (1.840-2.104) | 1.753 (1.639-1.875) |
| ALD | 6,480 | 1,020 | 33224.24 | 30.70 | 1.995 (1.869-2.129) | 2.223 (2.080-2.375) | 1.898 (1.775-2.029) |

Model 1 was unadjusted.

Model 2 was adjusted for age and sex.

Model 3 was adjusted for age, sex, low-income level, smoking status, regular exercise, CCI score, and hypertension, dyslipidemia and chronic kidney disease.

Abbreviations: DM diabetes mellitus; SLD steatotic liver disease; AF atrial fibrillation; IR incidence rate, PY person-years; HR hazard ratio; CI confidence interval; MASLD metabolic dysfunction-associated steatotic liver disease; MetALD metabolic dysfunction-associated steatotic liver disease with increased alcohol intake; ALD alcohol-associated liver disease; CCI Charlson comorbidity index.

**Supplementary Table 6.** Sensitivity analysis: the risk of incident DM across different SLD groups in patients with AF according to age strata applying a 2-year lag for DM onset.

| Age | SLD group | No. of patients | DM cases | Follow-up duration (PY) | IR (1000 PY) | aHR (95% CI) |
| --- | --- | --- | --- | --- | --- | --- |
|  |  |  |  |  |  | **Model 3** |
| 20-39 |  |  |  |  |  |  |
|  | **non-SLD** | 4,899 | 66 | 31336.35 | 2.11 | 1 (Ref.) |
|  | **MASLD** | 2,618 | 227 | 15722.74 | 14.44 | 5.893 (4.478-7.756) |
|  | **MetALD** | 477 | 38 | 2819.40 | 13.48 | 5.202 (3.488-7.758) |
|  | **ALD** | 270 | 28 | 1582.69 | 17.69 | 6.736 (4.327-10.485) |
| 40-49 |  |  |  |  |  |  |
|  | **non-SLD** | 9,340 | 373 | 57493.30 | 6.49 | 1 (Ref.) |
|  | **MASLD** | 6,415 | 814 | 36500.22 | 22.30 | 3.065 (2.709-3.468) |
|  | **MetALD** | 1,273 | 158 | 7209.95 | 21.91 | 2.869 (2.379-3.460) |
|  | **ALD** | 912 | 132 | 5054.67 | 26.11 | 3.171 (2.597-3.872) |
| 50-59 |  |  |  |  |  |  |
|  | **non-SLD** | 19,149 | 1,266 | 113926.98 | 11.11 | 1 (Ref.) |
|  | **MASLD** | 14,457 | 2,224 | 80512.42 | 27.62 | 2.289 (2.134-2.455) |
|  | **MetALD** | 2,304 | 310 | 12731.90 | 24.35 | 1.972 (1.740-2.236) |
|  | **ALD** | 1,837 | 330 | 9898.62 | 33.34 | 2.551 (2.257-2.883) |
| 60-69 |  |  |  |  |  |  |
|  | **non-SLD** | 27,139 | 2,510 | 150194.45 | 16.71 | 1 (Ref.) |
|  | **MASLD** | 20,261 | 3,612 | 106265.37 | 33.99 | 1.923 (1.826-2.024) |
|  | **MetALD** | 2,007 | 301 | 10686.99 | 28.17 | 1.587 (1.407-1.790) |
|  | **ALD** | 1,898 | 331 | 9701.81 | 34.12 | 1.842 (1.641-2.068) |
| 70-79 |  |  |  |  |  |  |
|  | **non-SLD** | 31,172 | 3,342 | 153655.63 | 21.75 | 1 (Ref.) |
|  | **MASLD** | 19,394 | 3,389 | 91733.60 | 36.94 | 1.626 (1.549-1.706) |
|  | **MetALD** | 1,139 | 192 | 5330.00 | 36.02 | 1.661 (1.435-1.923) |
|  | **ALD** | 1,336 | 181 | 6203.08 | 29.18 | 1.259 (1.084-1.463) |
| ≥80 |  |  |  |  |  |  |
|  | **non-SLD** | 12,622 | 963 | 46112.07 | 20.88 | 1 (Ref.) |
|  | **MASLD** | 5,264 | 670 | 19207.48 | 34.88 | 1.584 (1.435-1.748) |
|  | **MetALD** | 170 | 16 | 611.36 | 26.17 | 1.292 (0.788-2.118) |
|  | **ALD** | 227 | 18 | 783.36 | 22.98 | 1.062 (0.666-1.693) |
|  |  |  |  |  |  | p-for-interaction <.0001 |

Model 3 was adjusted for age, sex, low-income level, smoking status, regular exercise, CCI score, hypertension, dyslipidemia and chronic kidney disease.

Abbreviations: DM diabetes mellitus; SLD steatotic liver disease; AF atrial fibrillation; IR incidence rate, PY person-years; aHR adjusted hazard ratio; CI confidence interval; MASLD metabolic dysfunction-associated steatotic liver disease; MetALD metabolic dysfunction-associated steatotic liver disease with increased alcohol intake; ALD alcohol-associated liver disease; CCI Charlson comorbidity index.

**Supplementary Figure 1**. Study flow for sensitivity analysis using SLD definition of FLI ≥60.


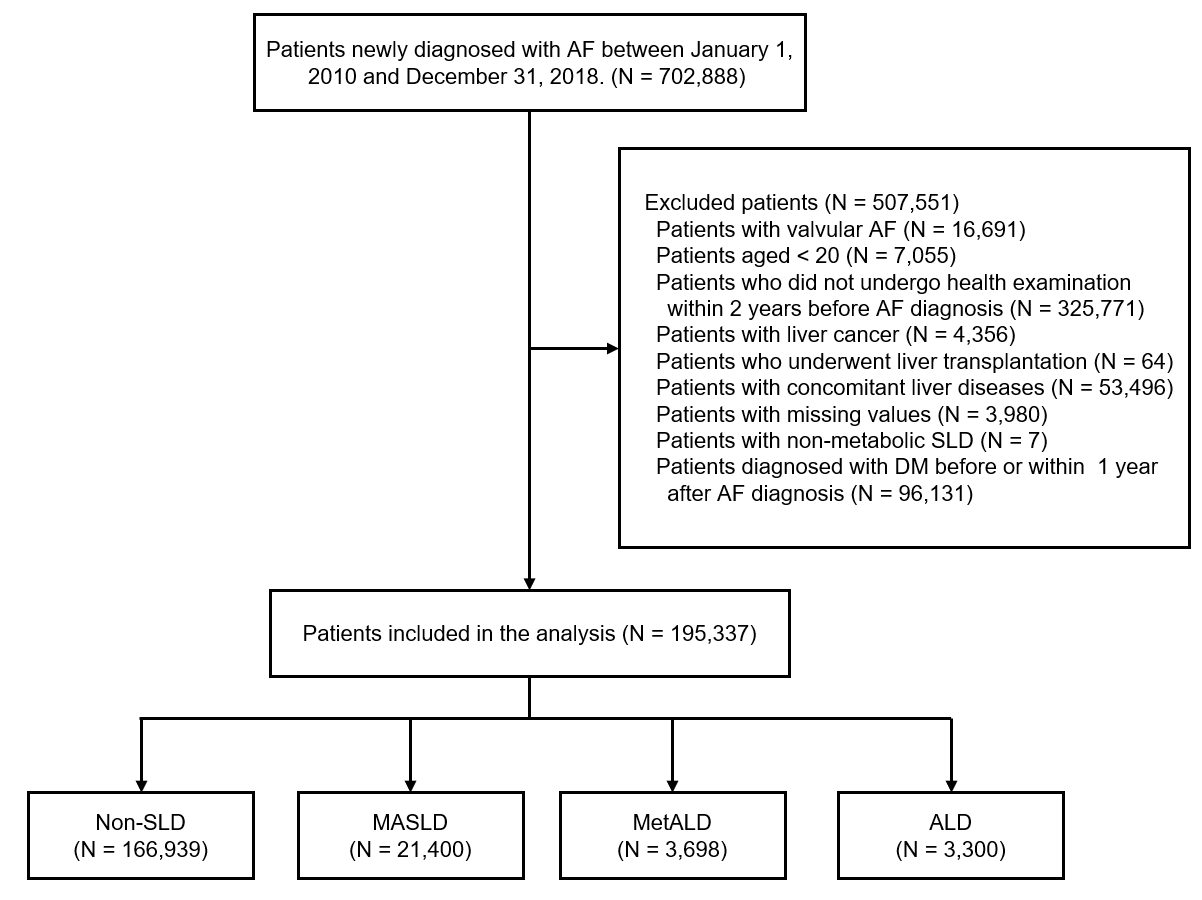


Abbreviations: AF atrial fibrillation; SLD steatotic liver disease; DM diabetes mellitus; MASLD metabolic dysfunction-associated steatotic liver disease; MetALD metabolic dysfunction-associated steatotic liver disease with increased alcohol intake; ALD alcohol-related liver disease; FLI fatty liver index.

**Supplementary Figure 2**. Study flow for sensitivity analysis applying a 2-year lag for DM onset.


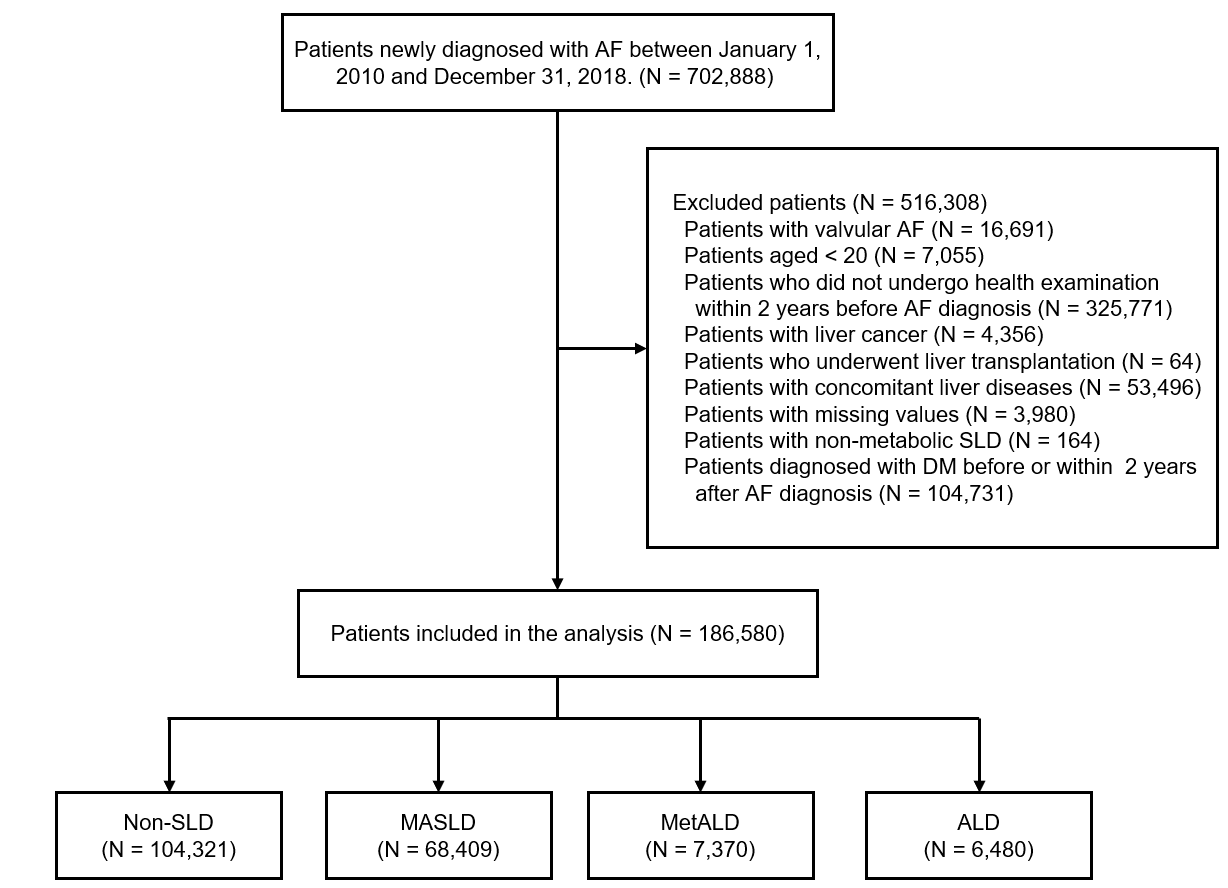


Abbreviations: DM diabetes mellitus; AF atrial fibrillation; SLD steatotic liver disease; MASLD metabolic dysfunction-associated steatotic liver disease; MetALD metabolic dysfunction-associated steatotic liver disease with increased alcohol intake; ALD alcohol-related liver disease; FLI fatty liver index.
